# California State Veterinary Medical Association

**Published:** 1892-08

**Authors:** R. C. Archibald

**Affiliations:** Sec’y.; Sacramento, Cal.


### SOCIETY PROCEEDINGS.

---

*California State Veterinary Medical Association.*—The Fifteenth Regular Meeting of the California State Veterinary Medical Association, was held at the Baldwin Hotel, San Francisco, June 9, 1892.

The meeting was called to order by the President, Dr. W. E. D. Morrison of Los Angeles. The roll being called, the following gentlemen responded : Drs. Maclay, Spencer, Sr., Spencer, Jr., Masoero, Witherspoon, Purce, Parent, Fox, Morrison, Wadams, Archibald, Orvis, Eagan, Davenport and Lazug. Visitors : Drs. Neif, Skaife and Ryke.

The minutes of the previous meeting were read and approved. Letters of regret were read from absent members.

The president then read his inaugural address, his subject being "Etiquette and Harmony," as follows :

Mr. President and Gentlemen :—A subject for an inaugural address to a society like ours, is not of easy choice. Unless one is naturally gifted with silver-tongued oratory, it is a difficult matter to render an address that shall at the same time be pleasing to the animal senses and still contain food for the mind to ponder and digest, so that the nutrition derived from the mental digestive process may benefit the whole social body. Milton said: that "Fools rush in where angels fear to tread," but I hope that my precautious folly may partake somewhat of the angelic caution, so that my subjects will not offend, but will tend to draw us all together in bonds more fraternally professional for our mutual and professional benefit.

The subjects I have chosen are etiquette and harmony, two, in my humble opinion, of the most importance to the veterinary profession. Etiquette should be observed with the nicest accuracy in our dealings one with the other. Each individual should consider the other individual's professional feelings. There should be no trifling with another's reputation, either by direct or indirect imputation. No sneering remarks or half uttered expressions, leading outsiders to draw detrimental influences. It is not always what is said, but what is unsaid, by saying. "I would rather not discuss the matter," thus conveying the idea that there is either malpractice or poor practice exercised by a fellow practitioner. So the seeds of doubt are sown in the client's mind, and thus casting discredit on the whole profession. The client will say, "That doctor knows not his business." The one to whom this is said will often answer, especially if he be a would-be veterinary surgeon, or local authority on diseases of the equine, bovine or canine, "Oh, they are all alike, they don't know any more than we do. I can cure colic or bots as well as any of them." Thus it goes, as we all well know. By this simple every day experience of all of us, I wish to demonstrate and impress on you the fact, how easy it is to destroy and how difficult to restore a professional reputation. It is as delicate as the most beautiful hot house flower. Handle it with care and natural caution and it lasts its natural life. Give it the least rough handling and its bloom is shed never to return. How many times a competent veterinary surgeon, a man that the profession recognizes as one above mediocrity, struggles along simply because on his first few cases death unavoidably stepped in and robbed him of his reputation.

On the other hand, another practitioner, who is purely superficial, but suave and sweet to every man in his dealings with the public, climbs the ladder of success and falsely wears the laurel crown that belongs in justice to his poorer fellow practitioner. I do not wish to convey the idea that he "falls like Lucifer, never to rise again," but that he whose reputation becomes injured from any cause, must of necessity work harder and suffer taunts of predjudice and ignorance until he regains the height from which he fell. Possessing the merit, it is only a question of time when he must succeed and wrest from the pretender his wrongfully obtained laurels. Mr President, the code of ethics of this Association covers completely the ground that I would have each member stand firmly on. We can in session formulate rules and regulations, but they are useless, unless each one truly and earnestly makes up his mind to carry out the code, not only in act but in kindly professional spirit.

It is also essential to the well being of the veterinary profession that harmony reign in our midst and that white winged peace prevail in our councils. United in the harmonious bonds of earnest professional sympathy, we can successfully resist our two most baneful enemies—ignorance, and its offspring, quackery. We must present a solid front to our enemies. There must be no dissensions or traitors in our ranks. If I may be allowed to paraphrase the old Biblical saying, that a profession divided against itself cannot stand. If jealousy and internal quarrels are to prevail, we do but weaken our professional position, and our councils come to naught. Division, instead of unity, is what quackery and our enemies most desire. It is the only possible method that gives them an opportunity to strike a fatal blow at the veterinary profession. This division in the profession in this State has already cost, us on two occasions, the passage of our bills to regulate the practice of veterinary medicine, and at the same time this division has given birth to another veterinary society which has no real cause for existence and never had Instead of this dog-in-the-manger policy, how far superior united policy would have been. Instead of two veterinary committees trying to exterminate each other in Sacramento; bidding against each other for votes; endeavoring to impress their different views on the representatives of this State, how much more professional, with what a stronger and more impressive voice we could have demanded attention to our wants; what greater respect we should have commanded as a united profession, aiming to benefit the health and stock interests of this State. Instead of two factions striving to ob tain what must have appeared to the representatives a slice of spoils. Two bills for a State Veterinarian and his assistants, two bills to regulate the practice of Veterinary Medicine—just ponder over it—and there are so few of us. What conclusions could they draw? "Horse doctors scrambling for boodle," as I heard a highly intelligent gentleman describe our Kilkenny-cat-like lobbying. It must of necessity have appeared in this light to the public. Mr. President, this endless suicidal strife must cease if we would succeed and raise our profession to the same level as the human profession. It is true we stand socially higher than we did ten years ago, but that is no good or sufficient reason why we should relax our vigilance or cease in the good work. Onward and upward we must press our veterinary banner, flinging its broad folds to the wind and inscribing in flaming letters on its broad surface the motto, "Excelsior." Mr. President and brother practitioners, let us each go forth from this meeting with a determination to fight the good fight, to stand shoulder to shoulder in the good cause to help instead of undoing a brother practitioner, and when our little race is run we shall have the satisfaction of knowing that we have borne ourselves bravely in the scientific fight against empircism and that we have left "footsteps in the sands of time" that shall be a warning as well as an encouragement; a warning against our enemies, an encouragement to follow our examples.

Dr. Parent then read a very instructive and interesting essay on "Wounds of Articular and Bursal cavities.

At the close of the paper he stated as to his treatment of the above named wounds as follows:

"When an accident results in the opening of a bursæ, or articular cavity, the exposure of the surface, together with the escape of synovia, will produce a high state of inflammation. The great secret then is to close the opening made as soon as possible, which while restoring the integrity of the cavity and stopping the discharge of synovia, will also stop the inflammation. It very often happens that we are not called till several days after the accident occurred and injudicious means have been employed; we then find the parts swollen, hot and tender in the extreme, together with high symptomatic fever. It is then necessary that the patient be treated constitutionally and locally until the inflammation becomes abated, adhesive inflammation is to be taken advantage of, as by throwing out coagulable lymph; the edges of the orifice may become united, thus preventing the escape of the synovial fluid. The necessity of this being attempted by artificial means is made imperative, because it is seldom that nature alone can secure that end. Our next enquiry is into the means of raising the proper amount of adhesive inflammation. This was formerly done by the use of the firing-iron or the application of some very strong blister, the object in view being to produce a scab for granulations to form under. Some practitioners after using the iron apply a blister over the part, thinking that they thereby lessened the secretion of synovia. The method I have employed with the greatest amount of success, is after having thoroughly cleansed the wound of all dirt, gravel or other foreign substances, to place my patient in such a position with slings, neck-cradle and splints. that he must keep perfectly quiet, then apply to the wound the following mixture in the form of a paste: Solid extract of belladonna one-half ounce, tanic acid one ounce, carbolic acid one drachm, wheat flour two ounces; mix with water into a paste, and apply as a plug to the opening. If possible place over all a cold poultice. With this treatment persisted in I have been successful in setting up fine granulations and closing the opening, where not too large, in from five to seven days. I have also, when I have found that I could not keep the plug in position, been fortunate in the use of oil of cloves painted into the wound two or three times a day."

The Secretary then read an essay on Actinomycosis. A discussion followed.

Dr. Maclay mentioned a case of Trichinosis, which the man had contracted from eating pork affected with Trichinæ.

The subject matter of legislation was brought up and thoroughly discussed, and on motion by Dr. Maclay. Drs Fox, Spencer, Jr., and the secretary were appointed a committee of three to draw up a bill by next meetlng. Carried.

It was moved by Dr. Maclay that the Secretary notify the public through the columns of the *Breeder and Sportsman* that upon application to the Association they will endeavor to procure qualified veterinarians for any district capable of supporting a qualified man. Carried.

On motion, a vote of thanks was tendered to the President and Essayists for the able manner which they had entertained the meeting.

The following gentlemen were admitted as members: Dr. R. D. Davidson, San Bernardino, and Dr. Mef, of San Francisco.

The names of the following gentlemen were proposed for membership: Dr. J. C. C. Price of Los Angeles, and Dr. F. W. Skaife of San Francisco.

The President appointed Drs. Eagan, Davenport and Purce as essayists for next meeting. After the transaction of some further business the meeting adjourned to meet in San Francisco on September 7, 1892.

R. C. ARCHIBALD, *Sec'y.*

Sacramento, Cal.

